# Performance of Convolutional Neural Network Models in Meningioma Segmentation in Magnetic Resonance Imaging: A Systematic Review and Meta-Analysis

**DOI:** 10.1007/s12021-024-09704-3

**Published:** 2024-12-28

**Authors:** Ting-Wei Wang, Jia-Sheng Hong, Wei-Kai Lee, Yi-Hui Lin, Huai-Che Yang, Cheng-Chia Lee, Hung-Chieh Chen, Hsiu-Mei Wu, Weir Chiang You, Yu-Te Wu

**Affiliations:** 1 Institute of Biophotonics, National Yang Ming Chiao Tung University, 155, Sec. 2, Li-Nong St. Beitou Dist, Taipei, 112304 Taiwan; 2 School of Medicine, College of Medicine, National Yang Ming Chiao Tung University, Taipei, 112304 Taiwan; 3 Department of Computer Science, Whiting School of Engineering, Johns Hopkins University, Baltimore, MD USA; 4 Department of Radiation Oncology, Taichung Veterans General Hospital, Taichung, 407219 Taiwan; 5 College of Computer Science, National Yang Ming Chiao Tung University, Hsinchu, 300093 Taiwan; 6 Department of Neurosurgery, Neurological Institute, Taipei Veterans General Hospital, Taipei, 112201 Taiwan; 7 Department of Radiology, Taichung Veterans General Hospital, Taichung, 407219 Taiwan; 8 Department of Radiology, Taipei Veterans General Hospital, Taipei, 112201 Taiwan

**Keywords:** Convolutional neural networks, MRI, Meningioma, Segmentation, Systematic review, Meta-analysis

## Abstract

**Background:**

Meningioma, the most common primary brain tumor, presents significant challenges in MRI-based diagnosis and treatment planning due to its diverse manifestations. Convolutional Neural Networks (CNNs) have shown promise in improving the accuracy and efficiency of meningioma segmentation from MRI scans. This systematic review and meta-analysis assess the effectiveness of CNN models in segmenting meningioma using MRI.

**Methods:**

Following the PRISMA guidelines, we searched PubMed, Embase, and Web of Science from their inception to December 20, 2023, to identify studies that used CNN models for meningioma segmentation in MRI. Methodological quality of the included studies was assessed using the CLAIM and QUADAS-2 tools. The primary variable was segmentation accuracy, which was evaluated using the Sørensen–Dice coefficient. Meta-analysis, subgroup analysis, and meta-regression were performed to investigate the effects of MRI sequence, CNN architecture, and training dataset size on model performance.

**Results:**

Nine studies, comprising 4,828 patients, were included in the analysis. The pooled Dice score across all studies was 89% (95% CI: 87–90%). Internal validation studies yielded a pooled Dice score of 88% (95% CI: 85–91%), while external validation studies reported a pooled Dice score of 89% (95% CI: 88–90%). Models trained on multiple MRI sequences consistently outperformed those trained on single sequences. Meta-regression indicated that training dataset size did not significantly influence segmentation accuracy.

**Conclusion:**

CNN models are highly effective for meningioma segmentation in MRI, particularly during the use of diverse datasets from multiple MRI sequences. This finding highlights the importance of data quality and imaging sequence selection in the development of CNN models. Standardization of MRI data acquisition and preprocessing may improve the performance of CNN models, thereby facilitating their clinical adoption for the optimal diagnosis and treatment of meningioma.

**Supplementary Information:**

The online version contains supplementary material available at 10.1007/s12021-024-09704-3.

## Introduction

Meningioma, the most common type of primary brain tumor, originates from the meningeal layers that envelop the brain and spinal cord. This malignancy accounts for approximately 37.6% of all cases of primary brain tumors (Ostrom et al. 2017). Meningioma can develop anywhere within the cranial or spinal meninges, manifesting as various clinical presentations. Most patients develop a benign form of meningioma (World Health Organization [WHO] Grade 1); however, a small proportion of patients develop atypical (WHO Grade 2) or malignant (WHO Grade 3) histological features (Louis et al. 2016). Despite its predominantly benign nature, meningioma can compress critical brain structures during tumor growth, resulting in various neurological symptoms and potentially life-threatening complications. This phenomenon underscores the urgent need for timely and accurate diagnosis as well as strategic treatment planning (Goldbrunner et al. 2016).

Magnetic resonance imaging (MRI) is the predominant tool for the detection and management of meningioma. The superior soft tissue contrast in MRI facilitates a comprehensive evaluation of tumor size, location, and interface, with adjacent brain or spinal cord structures. This imaging modality is indispensable for not only initial diagnosis but also surgical planning, treatment response monitoring, and tumor growth surveillance in patients not requiring immediate intervention (Whittle et al. 2004). MRI helps differentiate meningioma from other intracranial neoplasms and neurological conditions, thereby facilitating an accurate diagnosis, which is crucial for selecting the most appropriate therapeutic approach (Alexiou et al. 2010).

The accurate segmentation of meningioma in MRI—a vital step in tumor volume estimation and treatment planning—is difficult. These challenges stem from the tumor’s variable appearance on MRI: heterogeneity in signal intensity and presence of adjacent structures or edema that may obscure tumor boundaries. This variability necessitates sophisticated analytical techniques for reliable differentiation of meningioma from surrounding tissues (Clark et al. 2013). Traditionally performed by radiologists, manual segmentation is time-consuming approach subject to variability in interpretation. These limitations have spurred interest in automated segmentation methods, with convolutional neural network (CNN) models emerging as promising tools for this purpose. By leveraging deep learning (DL) algorithms, CNNs can markedly improve the accuracy, efficiency, and reproducibility of meningioma segmentation in MRI (Havaei et al. 2017).

The advent of CNN models and their application in medical imaging represent a pivotal shift toward the automation of complex diagnostic tasks. Owing to their ability to learn from extensive imaging data, CNN models are becoming increasingly adept at identifying subtle patterns and features indicative of specific pathologies, including meningioma. The application of these models in meningioma MRI segmentation promises to streamline the diagnostic workflow and refine treatment planning, ultimately enhancing clinical outcomes (Menze et al. 2015).

Given these advances, systematic reviews and meta-analyses on the performance of CNN models in meningioma MRI segmentation are required. Previous systematic reviews and meta-analyses have focused on general brain tumors, malignant gliomas, and brain metastases (Kouli et al. 2022; –Windisch et al. 2021). The present review focused on meningioma segmentation to synthesize current evidence, identify gaps in the literature, and steer future research toward the development of standardized, robust CNN models for this purpose. We primarily aimed to evaluate the accuracy, precision, and reliability of CNN models in meningioma MRI segmentation. For this, we assessed the methodological quality of relevant studies, ensuring the robustness of evidence and identifying factors that influence model performance—for example, CNN architecture, training dataset variability, and evaluation methods. In summary, this systematic review and meta-analysis was conducted to explore the applications of current CNN models in meningioma MRI segmentation and guide strategies for enhancing segmentation accuracy, efficiency, and standardization.

## Methods

### General Guidelines

This systematic review and meta-analysis adhered to the Preferred Reporting Items for Systematic Reviews and Meta-Analyses (PRISMA) 2020 guidelines (Page et al. 2021). The PRISMA guidelines were closely followed during the development and reporting stages to ensure methodological rigor. Additional details are presented in Supplementary Tables S1 and S2. The present study was formally registered in the PROSPERO database (identification number: CRD42023495352). Ethical approval and informed consent were deemed unnecessary because this study involved no human participants.

## Literature Search

Two reviewers, T-WW and H-JS, extensively searched the literature to identify studies using DL algorithms for meningioma segmentation in MRI. The reviewers searched PubMed, Embase, and Web of Science (Supplementary Table S2) from database inception to December 20, 2023. The process involved an initial screening of titles and abstracts, supplemented by a manual search to ensure exhaustive inclusion. A third reviewer with relevant expertise resolved any between-reviewer disagreements regarding study selection.

We included studies where meningioma in adult patients was diagnosed by applying DL algorithms on MRI data. We excluded non-English articles, retracted papers, studies using non-MRI or non-DL methods, irrelevant article types, duplicate datasets, studies reporting findings not pertinent to our research objective, and articles lacking data essential for meta-analysis (e.g., standard deviation values for Dice coefficients).

## Data Extraction and Management

T-WW and H-JS meticulously retrieved essential information from the included studies. Data on the following were collected: publication year, study design, study duration, patient demographics (age and sex), sample size, MRI series used in training and test datasets, bibliographic references, validation methods, data sources, indication standards, annotation software, magnetic field strength (measured in terms of Tesla), imaging sequences, skull stripping techniques, N4 bias field correction, image intensity normalization, image resolution adjustments, image augmentation techniques, image cropping, training dataset size, model dimensionality, and model algorithms.

We primarily used the Sørensen–Dice coefficient—a widely used metric crucial for evaluating the performance of segmentation algorithms.

## Methodological Quality Appraisal

T-WW and J-SH independently assessed the methodological quality of the included studies by using the Checklist for Artificial Intelligence in Medical Imaging (CLAIM) and Quality Assessment of Diagnostic Accuracy Studies-2 (QUADAS-2) tools (Mongan et al. 2020; Whiting et al. 2011). Between-reviewer discrepancies were resolved through discussion with experienced senior researchers until a consensus was achieved.

### Statistical Analysis

Two meta-analyses were conducted for the external validation group and the internal validation group to review studies that reported Dice coefficients. If a study reported multiple outcomes across different algorithms, the best-performing algorithm was selected for analysis. Furthermore, if multiple studies used the same dataset, the best outcome was selected for model validation. Data presented in terms of the median and interquartile range values were converted to the mean and standard deviation values (Wan et al. 2014; Luo et al. 2018). A random-effects model using restricted maximum likelihood estimation was used to account for the heterogeneity in study population (Borenstein et al. 2009). The results are depicted using forest plots.

Subgroup analyses (Borenstein and Higgins 2013) were performed to explore factors influencing model performance—for example, skull stripping, N4 bias field correction, intensity normalization, resolution adjustment, image augmentation, image cropping, MRI sequence type (single vs. multiple), imaging dimensionality (two-dimensional vs. two-dimensional/three-dimensional ensemble vs. three-dimensional imaging), and algorithm type (U-net vs. U-net variants vs. CNN). Meta-regression was performed to identify the associations between Dice scores and various factors, such as training dataset size (Morton 2004).

Heterogeneity among the included studies was evaluated using the Q test, with a *p* value of < 0.05 indicating statistical significance. Furthermore, heterogeneity was classified by *I*^2^values as trivial (0–25%), minimal (26–50%), moderate (51–75%), or pronounced (76–100%) (Higgins et al. 2003). Potential publication biases were investigated using the Egger test by examining funnel plot asymmetries (Egger et al. 1997). Statistical analyses were performed using Stata/SE (version 18.0; Texas, USA) for Mac.

## Results

### Study Selection

**Fig. 1:**
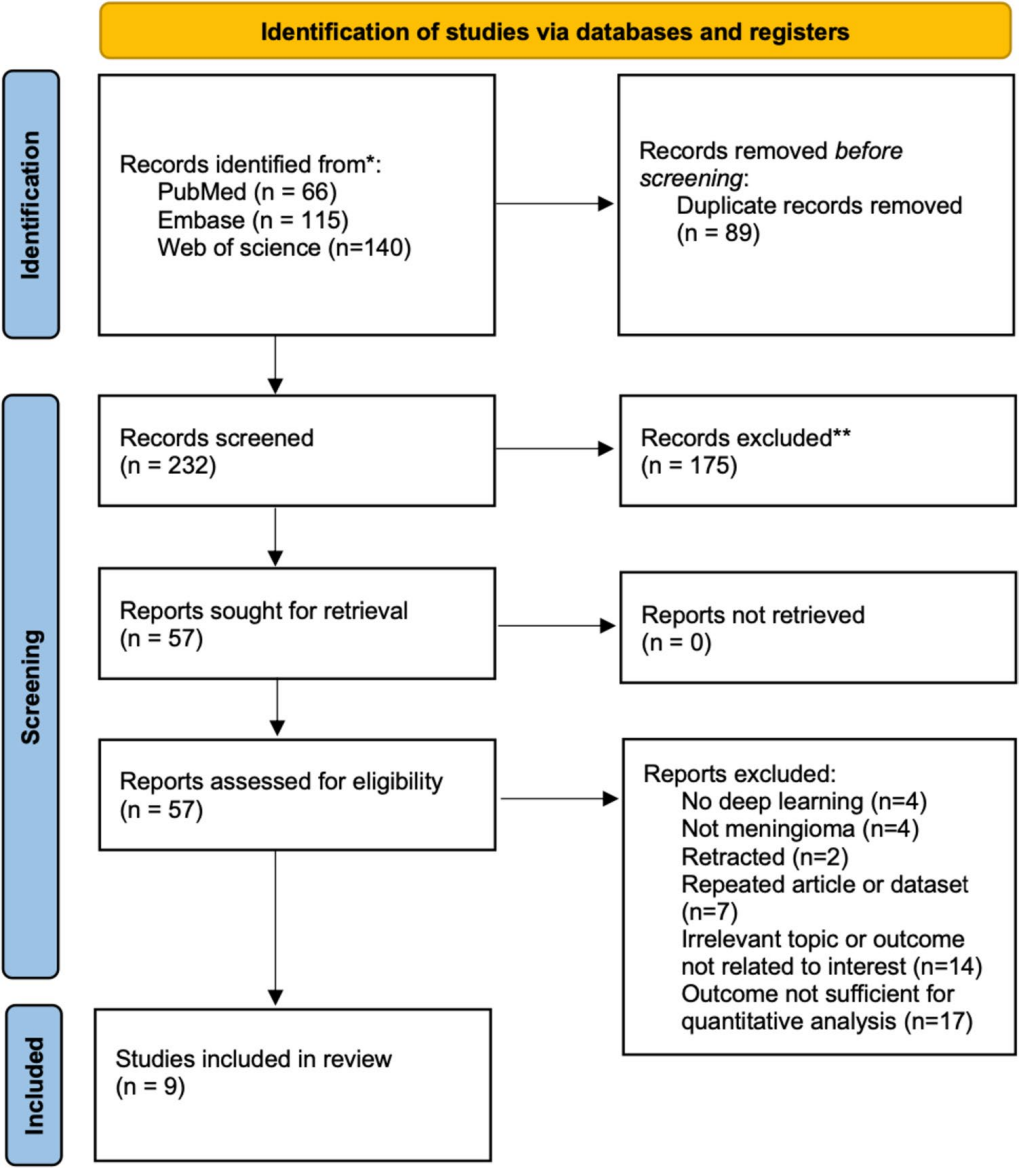
PRISMA flowchart for study selection

The literature search process is depicted in a PRISMA flowchart (Fig. 1). An initial query across multiple databases yielded 321 studies: 66 from PubMed, 115 from Embase, and 140 from Web of Science. After removing 89 duplicate entries, we used EndNote to sift through the remaining 232 articles. A screening of titles and abstracts led to the exclusion of an additional 175 articles because of their irrelevance to our study, per the study criteria. Further scrutiny of the remaining 57 full-text articles resulted in the exclusion of 48 articles (Sobhaninia et al. 2023; –Laukamp et al. 2018) because of them being datasets, news pieces, or conference presentations rather than empirical studies; not using DL algorithms; featuring repeated datasets; or covering topics outside the scope of our meta-analysis (Table S4). Finally, 9 suitable studies (Kang et al. 2023; Lee et al. 2023; –Laukamp et al. 2021) were included in the analysis.

## Basic Characteristics of the Included Studies

The characteristics of patients (*n* = 4,828) and methods used in the nine retrospective studies conducted between 2012 and 2022 are summarized in Table 1. The studies varied in terms of sample size (range: 91 patients (Hwang et al. 2022) to 1,069 patients (Lee et al. 2023)). Methodologically, all studies adopted a retrospective design, focusing on the analysis of pre-existing datasets. This approach is relevant for examining extensive patient history and outcome data over prolonged periods. The oldest dataset (2001) was obtained from a study by Kang et al. (2023) (Kang et al. 2023). Data analysis in these studies predominantly involved manual annotation, followed by validation by various experts, including experienced neurosurgeons, neuroradiologists, and radiologists; this step underscores the role of experts in interpreting medical image and outcome data.

**Table 1:** Patient and study characteristics

First author	Study design	Patients	Duration	Age	Sex (male)	Series (training/test)	Annotation	Validation	Data source	Indicator standard	Software
Lee et al. (2023)	Retrospective	1069	NR	NR	NR	1069(969/100)	Manual	Train/test	Taipei Veterans General Hospital	Experienced neurosurgeons and a neuroradiologist	GammaPlan system
Kang et al. (2023)	Retrospective	656	2001 ~ 2020	59.1(10.2)	551	659(459/200)	Manual	Train/test	Seoul National University College of Medicine	Neurosurgeons	MEDIP PRO
Jun et al. (2023)	Retrospective	318	2008 ~ 2018	59.5 (20.5)	80	318(257/61)	Manual	Train/test	Massachusetts General Hospital, Yonsei University College of Medicine	Neuroradiologist	3D slicer
Dong et al. (2023)	Retrospective	634	2018 ~ 2022	NR	NR	634(547/87)	Manual	Train/test	Jinling Hospital	Neuroradiologists	NR
Chen et al. (2023)	Retrospective	606	2015 ~ 2021	51.5 (9.3)	236	606(0/606)	Manual	Train/test	West China Hospital	Neuroradiologists	ITK-SNAP software
Hwang et al. (2022)	Retrospective	91	2016 ~ 2019	NR	NR	171(154/17)	Manual	Train/test	Seoul National University Bundang Hospital	Radiologists	NR
Chen et al. (2022)	Retrospective	609	2011 ~ 2019	51.51 (12.3)	169	609(307/302)	Manual	Train/test	Sun Yat-Sen University Cancer Centre, Xiangya Hospital Central South University	Readers	ITK-SNAP software
Bouget et al. (2022)	Retrospective	719	NR	NR	NR	719(577/142)	NR	NR	St. Olavs Hospital, Trondheim University Hospital	NR	NR
Laukamp et al. (2021)	Retrospective	126	2012 ~ 2018	58.4 (13.5)	75	127(70/56)	Manual	Train/test	University Hospitals Cleveland Medical Center	Radiologists	IntelliSpace Discovery

Abbreviations: *NR*, Not recorded

Most studies used train/test splits for result validation, indicating that this practice is standard in this subfield for evaluating model reliability and performance. The diversity of data sources across institutions such as Taipei Veterans General Hospital, Seoul National University College of Medicine, and Massachusetts General Hospital reflects the affiliations and the quality of data used. Various annotation software tools were used in the included studies—for example, the GammaPlan system, MEDIP PRO, 3D Slicer, and ITK-SNAP software; this indicates the varied and nature of the software tools used.

### MRI Characteristics, Preprocessing Techniques, and DL Algorithm Performance

The studies conducted from 2022 to 2023 focused on MRI scans at 1T, 1.5T, and 3T, particularly targeting T1-weighted, T1 contrast-enhanced, and T2-weighted, T2 FLAIR image sequences (Table 2). These studies emphasized the importance of various preprocessing steps in enhancing data quality for the application of DL models in medical imaging. Intensity normalization, which was widely performed across the included studies (Kang et al. 2023; Lee et al. 2023; –Laukamp et al. 2021), served as a technique for reducing scanner-induced variability and ensuring consistency across scans.

**Table 2:** MRI characteristics, preprocessing techniques, and deep learning model performance

First author	Tesla	Image sequence	Skull stripping	N4 bias field correction	Intensity normalization	Resolution adjustment	Image augmentation	Image cropping	Training size	Input dimension	Algorithms
Lee et al. (2023)	1.5T	T1c, T2w	No	No	Yes	No	Yes	Yes	969	3D	dual-pathway CNN
Kang et al. (2023)	1T/1.5T/3T	T1c	No	No	Yes	Yes	Yes	Yes	459	2D	nnU-Net
Jun et al. (2023)	1.5T/3T	T1c, T2w	Yes	Yes	Yes	Yes	Yes	No	257	3D	U-net
Dong et al. (2023)	3T	T1c	No	No	Yes	No	Yes	Yes	547	3D	DE-Uformer
Chen et al. (2023)	1.5T/3T	T1c	No	No	Yes	Yes	Yes	Yes	735	3D	DeepLab V3+
Hwang et al. (2022)	1.5T/3T	T1c, T2w, T1w, T2FLAIR	Yes	Yes	No	No	Yes	No	154	3D	U-net
Chen et al. (2022)	1.5T/3T	T1c, T2w, T1w	No	Yes	Yes	Yes	Yes	Yes	307	3D	modified attention U-Net
Bouget et al. (2022)	NR	T1c	Yes	No	Yes	Yes	Yes	No	577	3D	AGU-Net
Laukamp et al. (2021)	1T/1.5T/3T	T1c, T2FLAIR	Yes	Yes	Yes	Yes	No	No	70	3D	DeepMedic

Abbreviations: *NR*, Not record

Resolution adjustment was performed in several studies (Kang et al. 2023; Jun et al. 2023; –Laukamp et al. 2021)to refine image quality for precise analysis. In addition, skull stripping and N4 bias field correction were performed (Jun et al. 2023; Hwang et al. 2022; Bouget et al. 2022; Laukamp et al. 2021), although less uniformly. Skull stripping is performed to remove nonbrain tissue areas from MRI scans, whereas N4 bias field correction is performed to correct scanner-induced inconsistencies in intensity. These steps are crucial for preparing images for subsequent analysis and ensuring that DL models are trained on data that accurately represent the brain’s anatomy and pathological features without extraneous information. Image augmentation were performed consistently across the studies (Kang et al. 2023; Lee et al. 2023; –Laukamp et al. 2021). These preprocessing techniques are essential for the development of robust and efficient DL models.

The training datasets exhibited variability, ranging from as few as 83 images (Kang et al. 2023) to as many as 597 images (Dong et al. 2023). The included studies used a range of DL algorithms, ranging from models such as ACMINet, PA-Seg, and TISS-Net to models such as CNN, ResNet, and nnU-net. This variation highlights a wide array of potential approaches for addressing challenges in brain lesion segmentation and the tailored application of both preprocessing techniques and algorithmic solutions. Thus, attention to detail is crucial for addressing the specificities of MRI data analysis.

### Results of Quality Assessment

Figure S1presents the results of a quality assessment performed using the QUADAS-2 tool. Supplementary Table S5 presents the results of an analysis of potential biases and applicability concerns. One study (Lee et al. 2023) omitted interval derivation data; this omission might have affected the accuracy of data representation. Furthermore, two studies [77, 89] excluded some patients from the analyses, potentially limiting the generalizability and interpretive value of the results.

Supplementary Table S6 presents a detailed overview of nine studies evaluated using the CLAIM tool. The average CLAIM score was 30 (approximately 70%; standard deviation: 5.15), with scores ranging from 22 to 36 (maximum score: 42). The average scores for different sections of the CLAIM tool were as follows: title/abstract, 1.56/2 (78%); introduction, 2.00/2 (100%); methods, 19.89/28 (71%); results, 3.22/5 (64%); discussion, 2/2 (100%); and other information, 0.89/3 (30%). These results highlight the strengths of the included studies and reveal areas for improvement. Our findings emphasize the potential of advancing research on meningioma segmentation through the integration of DL models.

### Efficacy of DL model–based Segmentation of Meningioma in MRI: Internal Validation

**Fig. 2:**
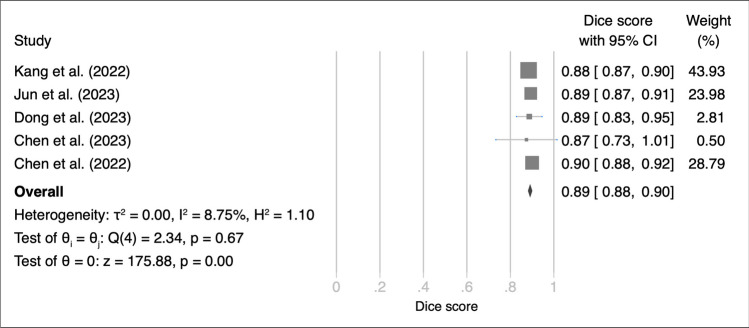
Forest plot of Dice scores for deep learning models used in meningioma segmentation with internal validation set

Our analysis included four studies detailing the application of various DL models for meningioma segmentation in internal validation dataset. This study revealed variations in Dice scores (range: 84–91%). An aggregated analysis yielded a pooled Dice score of 88% (95% confidence interval [CI]: 85–91%; Fig. 2). The Q test indicated significant heterogeneity among the included studies (Q = 95.39: *p* < 0.01). This result was further corroborated by a high Higgins *I*^2^ value (96.28%). Sensitivity analysis confirmed the robustness of these findings, suggesting that the summary effect sizes remained significant, even with the sequential exclusion of individual studies from the analysis (Figure S2).

**Fig. 3:**
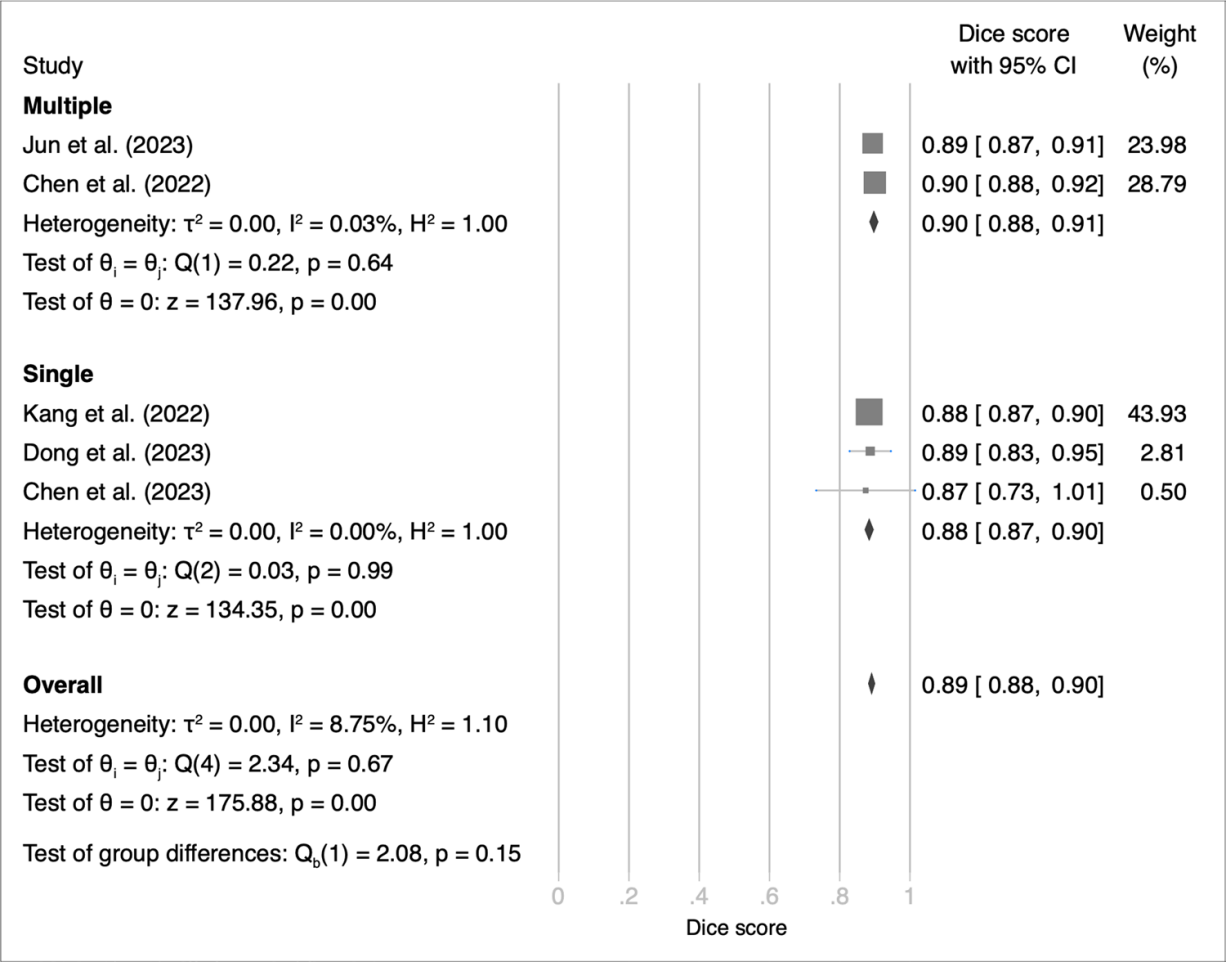
Subgroup analysis of image sequence showing Dice scores for deep learning models used in meningioma segmentation with internal validation set

We explored the sources of heterogeneity in the accuracy of various DL models in meningioma segmentation. For this, we performed subgroup analysis by MRI sequence type, which provided insights into the variability of model performance. The subgroup analysis also revealed a performance disparity between models trained on multiple MRI sequences and those trained on a single sequence (Fig. 3). Studies incorporating multiple MRI sequences achieved a pooled Dice score of 90% (95% CI: 90–90%), which indicated a high level of accuracy in meningioma segmentation. By contrast, studies using a single MRI sequence reported a pooled Dice score of 85% (95% CI: 84–86%).

Further subgroup analyses were performed to investigate the effects of various technical and methodological factors on segmentation performance. These factors included skull stripping techniques, N4 bias field correction, image intensity normalization, image resolution adjustments, image augmentation techniques, image cropping, and algorithm type. None of the indicated factors yielded significant differences in performance metrics (Figure S3).

Our meta-regression for the effect of training dataset size on the Dice score revealed no significant effect, indicating that the efficacy of DL models in meningioma segmentation does not rely solely on the quantity of training data. Furthermore, the funnel plot and subsequent Egger regression test (*p* = 0.46) indicated no significant publication bias in the included studies (Figure S4).

### Efficacy of DL model–based Segmentation of Meningioma in MRI: External Validation

**Fig. 4:**
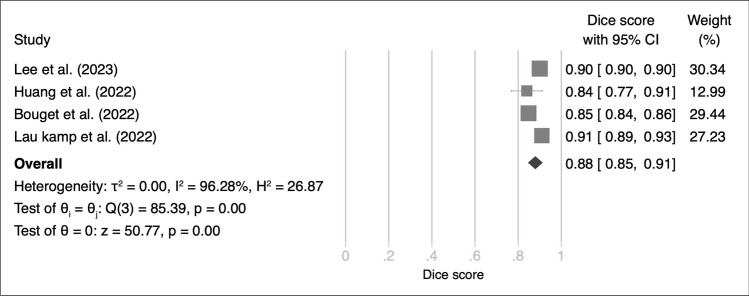
Forest plot of Dice scores for deep learning models used in meningioma segmentation with external validation set

We analyzed five studies that focused on applying various deep learning (DL) models for meningioma segmentation using external validation datasets. These studies demonstrated variations in Dice scores, which ranged from 87 to 90%. When we aggregated the results, the pooled Dice score was calculated to be 89% (95% CI of 88–90%) (Fig. 4).

The heterogeneity among the included studies was assessed using the Q test, which did not reveal any significant heterogeneity (Q = 2.34; *p* = 0.69). This finding was further supported by a low Higgins I² value of 8.75%, indicating minimal variability between studies. Sensitivity analysis reinforced the robustness of these results, showing that the overall effect sizes remained significant even when individual studies were sequentially excluded from the analysis (see Figure S5).

To explore potential sources of variation in segmentation performance, we conducted subgroup analyses examining various technical and methodological factors. These factors included image sequence, skull stripping techniques, N4 bias field correction, image resolution adjustments, image cropping, image dimension, and algorithm type. None of the indicated factors yielded significant differences in performance metrics (see Figure S6).

We performed a meta-regression to assess the effect of the training dataset size on the Dice score. The results indicated no significant impact, implying that the performance of DL models for meningioma segmentation is not solely dependent on the quantity of training data available. Furthermore, we evaluated the potential for publication bias using a funnel plot and conducted an Egger regression test. The test yielded a p-value of 0.96, indicating no significant publication bias among the included studies (refer to Figure S7). This adds credibility to our findings by suggesting that the results are representative and not influenced by selective reporting.

## Discussion

Our systematic review and meta-analysis underscore the high efficacy of deep learning (DL) models, particularly convolutional neural networks (CNNs), in the segmentation of meningiomas using magnetic resonance imaging (MRI). The pooled Dice coefficient from internal validation studies was 88% (95% confidence interval [CI]: 85–91%), while external validation studies yielded a pooled Dice coefficient of 89% (95% CI: 88–90%). These results demonstrate that DL models are proficient in accurately delineating meningioma boundaries, a critical factor in treatment planning and prognosis.

The variation in Dice scores across studies highlights the influence of different methodologies, datasets, and imaging protocols on model performance. Notably, our subgroup analysis revealed that models trained on multiple MRI sequences achieved higher accuracy than those utilizing a single sequence. In internal validation, models incorporating multiple MRI sequences attained a pooled Dice score of 90% (95% CI: 90–90%), whereas those relying on a single sequence had a pooled Dice score of 85% (95% CI: 84–86%). This finding underscores the importance of diverse imaging data in enhancing the performance of DL models.

Similarly, in external validation, the pooled Dice scores were consistent across studies, and the heterogeneity was minimal (I² = 8.75%). This minimal variability suggests that DL models generalize well across different external datasets, reinforcing their potential applicability in varied clinical settings.

Our analysis further highlighted significant differences between models using multiple MRI sequences and those relying on a single sequence. Subgroup analyses confirmed the superior accuracy of models trained on multiple MRI sequences, indicating that using multiple MRI sequences should become standard practice in DL-based meningioma segmentation. Importantly, the size of the training dataset did not significantly affect the Dice score, suggesting that data quality is more crucial than data volume for achieving accurate results. This highlights a key area for improvement in model training—by refining data preprocessing techniques and leveraging advanced CNN architectures that can learn effectively from complex imaging data.

Recent developments in the field support the growing potential of DL models for brain tumor segmentation. The Brain Tumor Segmentation (BraTS) Challenge 2023 (PMID: 37608937) (LaBella et al. 2023) has continued to advance algorithm development by providing a competitive platform for benchmarking state-of-the-art methods on standardized datasets. This challenge fosters collaboration and innovation, and the inclusion of comprehensive and diverse datasets including meningioma dataset encourages the creation of more robust and generalizable models, which is vital for translating these models into clinical practice.

In addition to CNNs, emerging technologies like Vision Transformers (ViTs) show promise in medical image analysis. ViTs use self-attention mechanisms to capture global contextual information, which can be particularly advantageous in handling complex and heterogeneous tumor structures (Dosovitskiy et al. 2020). Recent studies suggest that ViTs can outperform traditional CNNs in various segmentation tasks (Hatamizadeh et al. 2021). Incorporating ViTs into meningioma segmentation could further improve model performance by capturing long-range dependencies and complex features within MRI data.

However, several limitations should be considered. First, inherent biases in the design of the included studies, such as retrospective data collection and reliance on pre-existing datasets, could affect the generalizability of the findings. Second, while the Dice coefficient is a valuable metric for assessing segmentation accuracy, it might not fully capture performance variability across different complexity levels in segmentation tasks, possibly overlooking nuances in how models handle challenging or atypical image features. Lastly, the applicability of the findings may be limited by variations in MRI protocols or model architectures not covered in the reviewed studies.

Future research in DL-based meningioma segmentation should focus on several key areas. Standardizing imaging protocols and preprocessing techniques is crucial to improve model generalizability across different clinical settings. Optimizing the integration of multiple MRI sequences can enhance segmentation accuracy by providing comprehensive tumor information. Exploring advanced architectures, such as Vision Transformers, and comparing them with traditional CNNs may lead to improved model performance. Promoting data sharing and collaboration through initiatives like the BraTS Challenge will help develop more robust models by providing access to diverse, well-annotated datasets. Finally, research should prioritize the seamless integration of DL models into clinical workflows, evaluating user acceptance, assessing learning curves, and analyzing cost-effectiveness to support broader adoption in healthcare systems. These directions collectively aim to advance the field and enhance the clinical applicability of DL-based meningioma segmentation.

## Conclusion

Convolutional Neural Network (CNN) models demonstrate high effectiveness for meningioma segmentation in MRI, especially when utilizing datasets from diverse MRI sequences. This study underscores the crucial role of data quality and sequence selection in the development and performance of CNN models. Promoting standardization in MRI data acquisition and preprocessing may enhance CNN effectiveness, enabling better integration into clinical practice for optimal meningioma diagnosis and management.

## Supplementary Information

Below is the link to the electronic supplementary material.

ESM 1 (DOCX 15.1 MB)

## Data Availability

No datasets were generated or analysed during the current study.
