# Supplementary material for: Performance of Convolutional Neural Network Models in Meningioma Segmentation in Magnetic Resonance Imaging: A Systematic Review and Meta-Analysis
Source: Neuroinformatics. 2024 Dec 28;23(1):14. doi: 10.1007/s12021-024-09704-3 (PMC11706894; doi:10.1007/s12021-024-09704-3)
Supplement: Supplementary file 1 — (DOCX 15.1 MB) [file 12021_2024_9704_MOESM1_ESM.docx]

**Assessing the Performance of Convolutional Neural Networks in Meningioma MRI Segmentation: A Systematic Review and Meta-analysis**

**
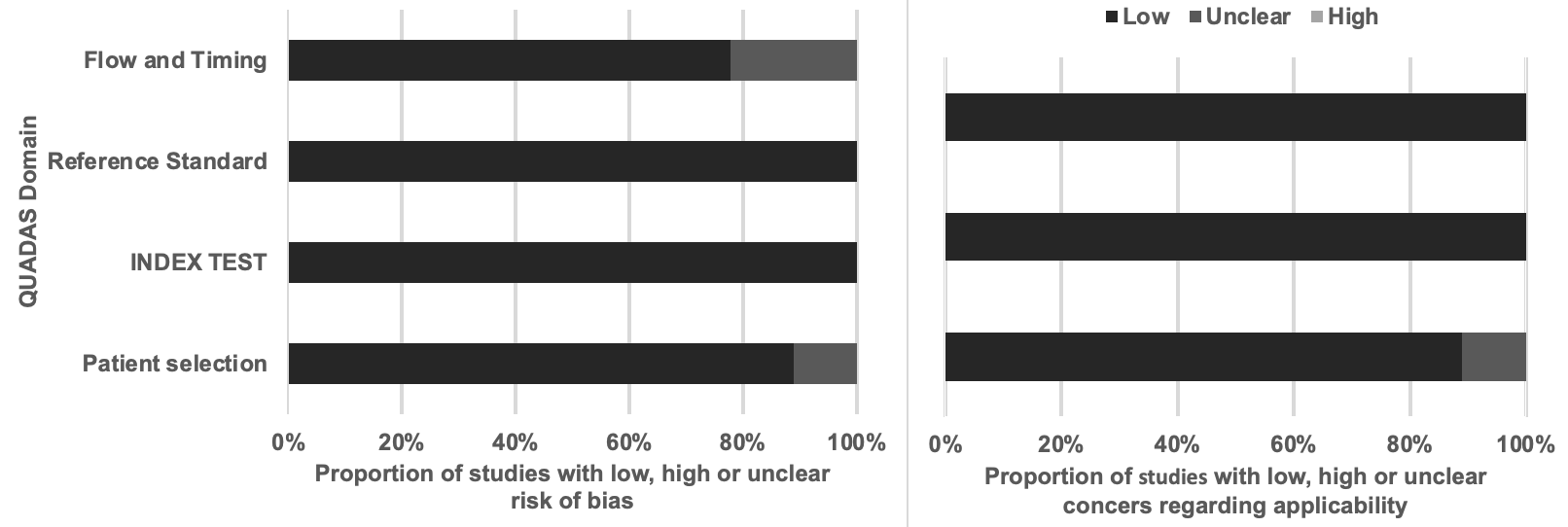
**

**Figure S1. The results of QUADAS-2 quality assessment for included studies.**


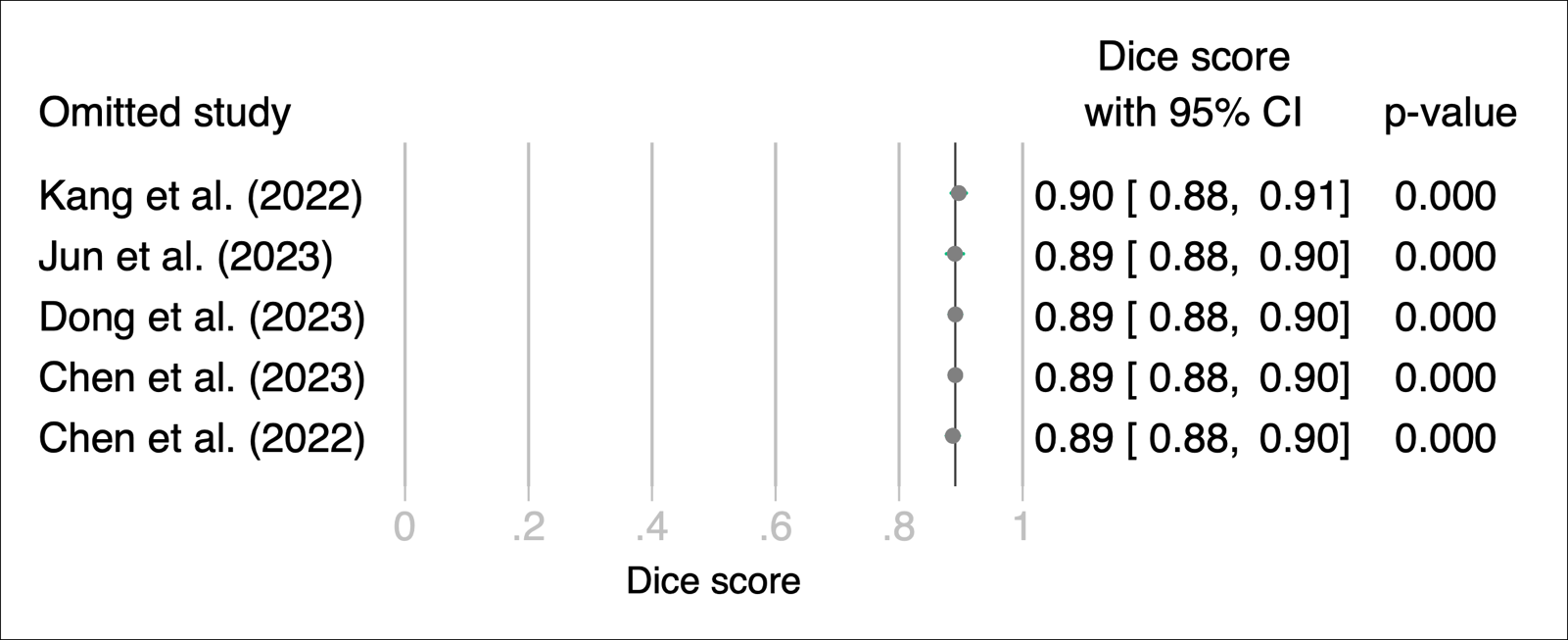


**Figure S2 The results of a sensitivity analysis of deep learning algorithms in segmenting meningioma with internal validation set.**


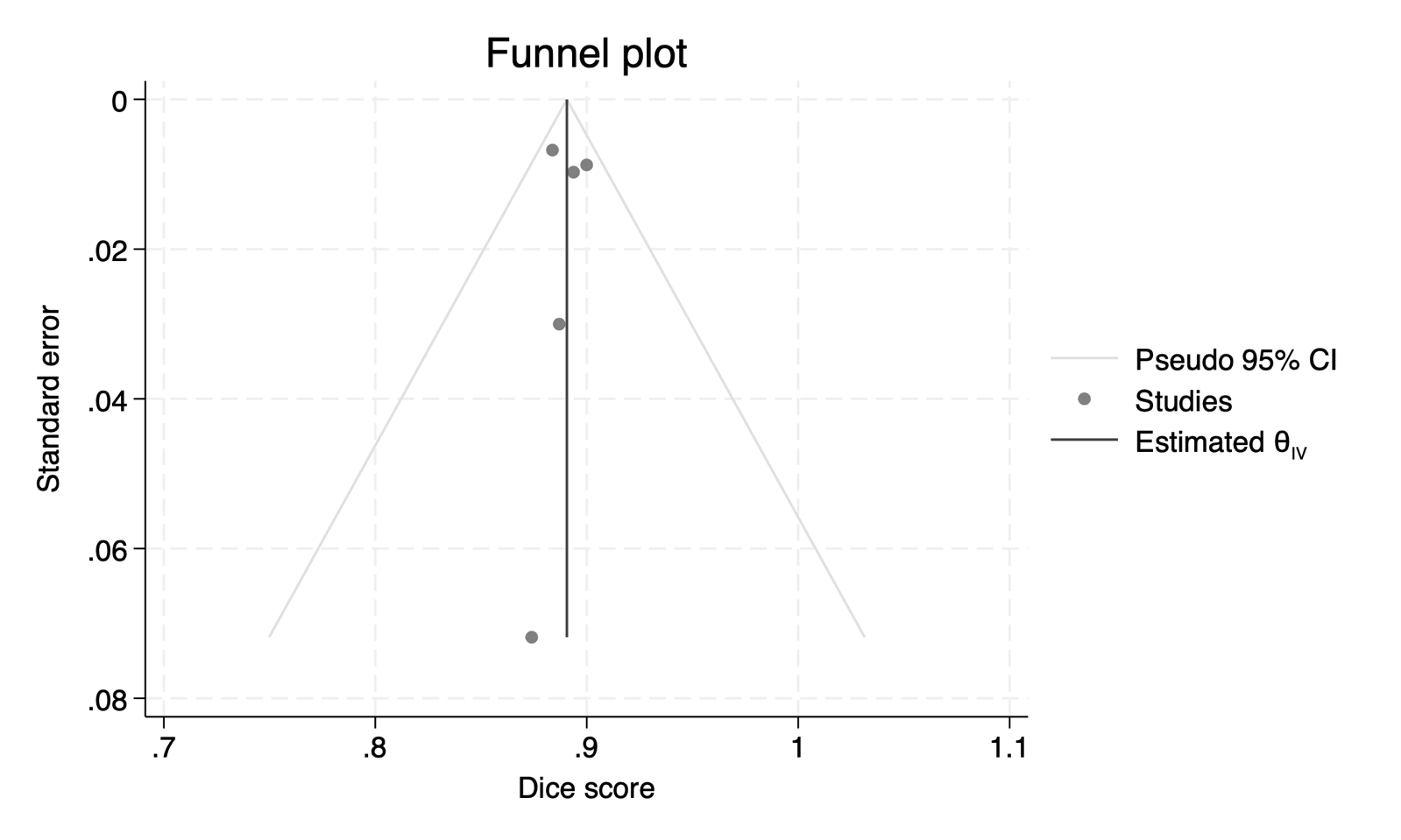


**Figure S3. The funnel plot of Dice scores for deep learning algorithms in segmenting meningioma with internal validation set.** The *p* value of the Egger's test was 0.4636 indicating no publication bias.

**
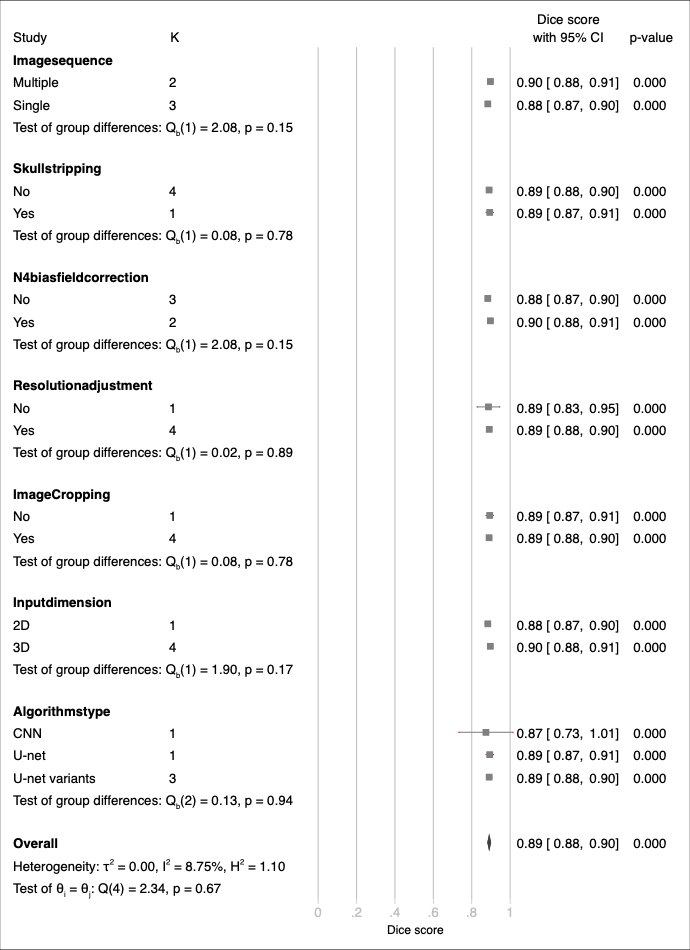
**

**Figure S4. Subgroup analysis of deep learning algorithms in segmenting meningioma with internal validation set.**

**
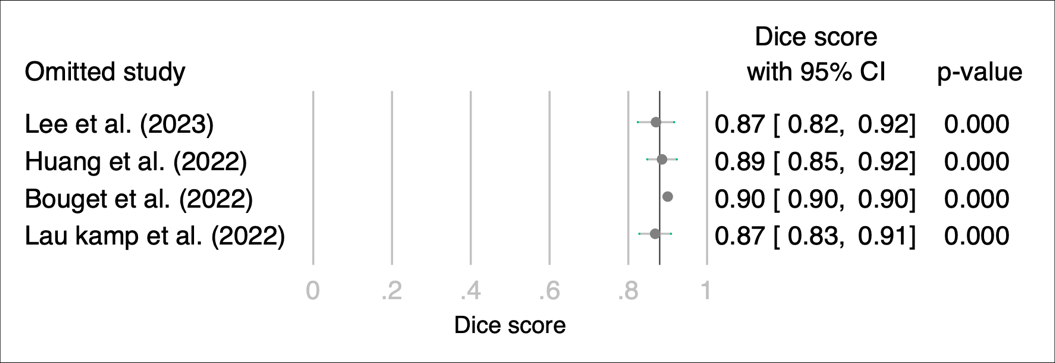
**

**Figure S5 The results of a sensitivity analysis of deep learning algorithms in segmenting meningioma with external validation set.**

**
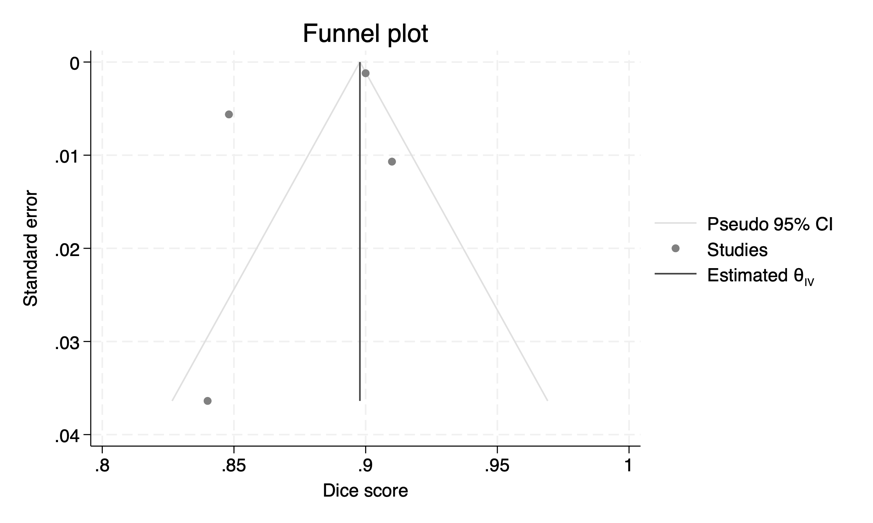
**

**Figure S6. The funnel plot of Dice scores for deep learning algorithms in segmenting meningioma with external validation set.** The *p* value of the Egger's test was 0.9649 indicating no publication bias.


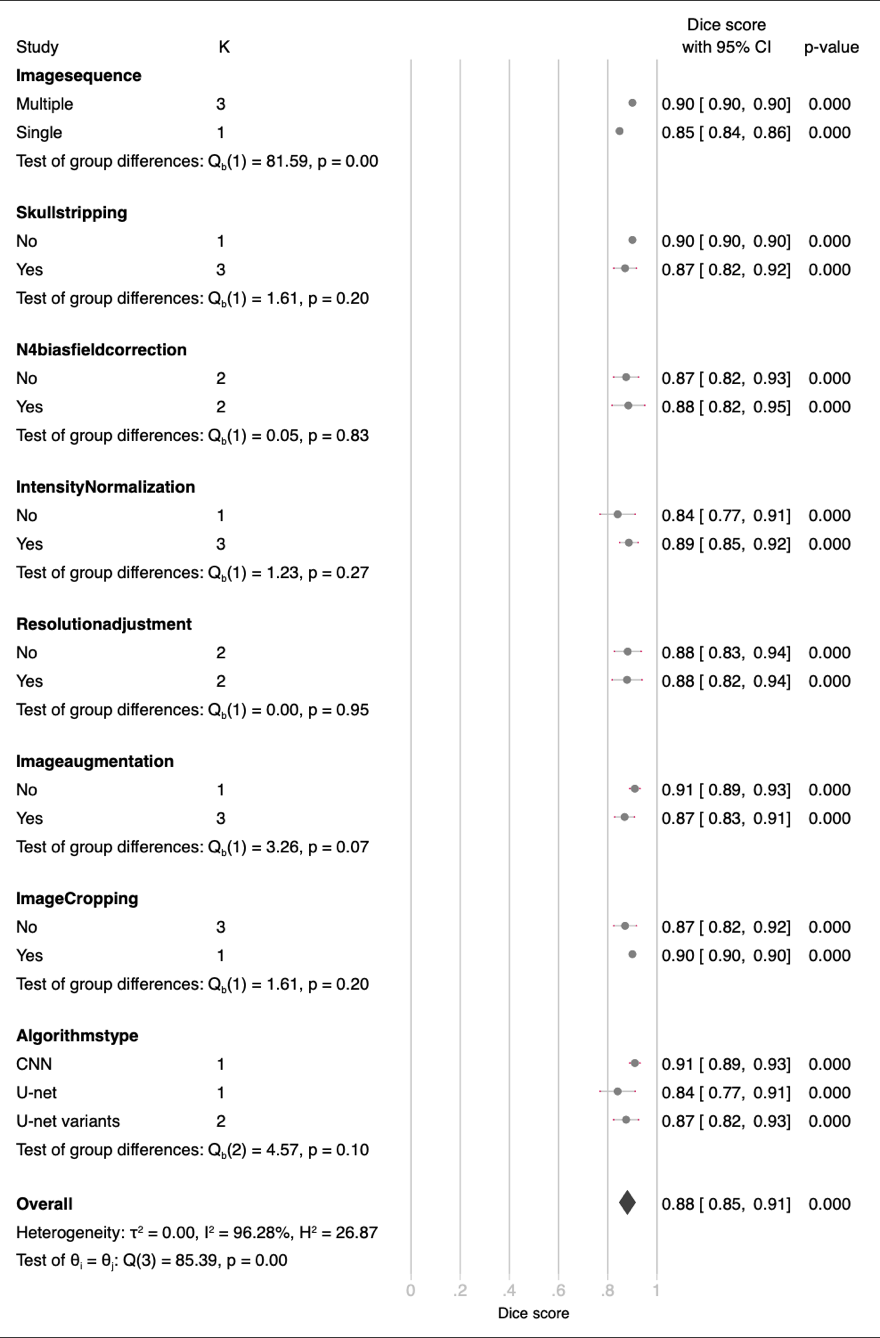


**Figure S7. Subgroup analysis of deep learning algorithms in segmenting meningioma with external validation set.**

**Table S1. PRISMA-DTA Abstract Checklist.**

| **Section/Topic** | **Number** | **PRISMA-DTA for Abstracts Checklist Item** | **Reported on Page #** |
| --- | --- | --- | --- |
| TITLE and PURPOSE | | | |
| Title | 1 | Identify the report as a systematic review (+/- meta-analysis) of diagnostic test accuracy (DTA) studies. | Title page |
| Objectives | 2 | Indicate the research question, including components such as participants, index test, and target conditions. | 1 |
| METHODS | | | |
| Eligibility criteria | 3 | Include study characteristics used as criteria for eligibility. | 1 |
| Information sources | 4 | List the key databases searched and the search dates. | 1 |
| Risk of bias & applicability | 5 | Indicate the methods of assessing risk of bias and applicability. | 1 |
| Synthesis of results | A1 |  | 1 |
| RESULTS | | | |
| Included studies | 6 | Indicate the number and type of included studies and the participants and relevant characteristics of the studies (including the reference standard). | 1 |
| Synthesis of results | 7 | Include the results for the analysis of diagnostic accuracy, preferably indicating the number of studies and participants. Describe test accuracy including variability; if meta-analysis was done, include summary results and confidence intervals. | 1 |
| DISCUSSION | | | |
| Strengths and limitations | 9 | Provide a brief summary of the strengths and limitations of the evidence | 2 |
| Interpretation. | 10 | Provide a general interpretation of the results and the important implications. | 2 |
| OTHER | | | |
| Funding | 11 | Indicate the primary source of funding for the review | NA |
| Registration | 12 | Provide the registration number and the registry name | NA |

Adapted From: McInnes MDF, Moher D, et al. The PRISMA-DTA Group (2018). Preferred Reporting Items for a Systematic Review and Meta-analysis of Diagnostic Test Accuracy Studies: The PRISMA-DTA Statement. JAMA. 2018 Jan 23;319(4):388-396. doi: 10.1001/jama.2017.19163.

**Table S2. PRISMA-DTA Checklist.**

| **Section/Topic** | **Number** | **PRISMA-DTA for Abstracts Checklist Item** | **Reported on Page #** |
| --- | --- | --- | --- |
| TITLE and PURPOSE | | | |
| Title | 1 | Identify the report as a systematic review (+/- meta-analysis) of diagnostic test accuracy (DTA) studies. | Title page |
| Abstract | 2 | Abstract: See PRISMA-DTA for abstracts. | 1-2 |
| INTRODUCTION | | | |
| Rationale | 3 | Describe the rationale for the review in the context of what is already known. | 2-5 |
| Clinical role of index test | D1 | State the scientific and clinical background, including the intended use and clinical role of the index test, and if applicable, the rationale for minimally acceptable test accuracy (or minimum difference in accuracy for comparative design). | 2-5 |
| Objectives | 4 | Provide an explicit statement of question(s) being addressed in terms of participants, index test(s), and target condition(s). | 2-5 |
| METHODS | | | |
| Protocol and registration | 5 | Indicate if a review protocol exists, if and where it can be accessed (e.g., Web address), and, if available, provide registration information including registration number. | 5 |
| Eligibility criteria | 6 | Specify study characteristics (participants, setting, index test(s), reference standard(s), target condition(s), and study design) and report characteristics (e.g., years considered, language, publication status) used as criteria for eligibility, giving rationale. | 5-6 |
| Information sources | 7 | Describe all information sources (e.g., databases with dates of coverage, contact with study authors to identify additional studies) in the search and date last searched. | 5-6 |
| Search | 8 | Present full search strategies for all electronic databases and other sources searched, including any limits used, such that they could be repeated. | 5-6 |
| Study selection | 9 | State the process for selecting studies (i.e., screening, eligibility, included in systematic review, and, if applicable, included in the meta-analysis). | 5-6 |
| Data collection process | 10 | Describe method of data extraction from reports (e.g., piloted forms, independently, in duplicate) and any processes for obtaining and confirming data from investigators. | 6 |
| Definitions for data extraction | 11 | Provide definitions used in data extraction and classifications of target condition(s), index test(s), reference standard(s) and other characteristics (e.g. study design, clinical setting). | 6 |
| Risk of bias and applicability | 12 | Describe methods used for assessing risk of bias in individual studies and concerns regarding the applicability to the review question. | 7 |
| Diagnostic accuracy measures | 13 | State the principal diagnostic accuracy measure(s) reported (e.g. sensitivity, specificity) and state the unit of assessment (e.g. per-patient, per-lesion). | 7-8 |
| Synthesis of results | 14 | Describe methods of handling data, combining results of studies and describing variability between studies. This could include, but is not limited to: a) handling of multiple definitions of target condition. b) handling of multiple thresholds of test positivity, c) handling multiple index test readers, d) handling of indeterminate test results, e) grouping and comparing tests, f) handling of different reference standards | 7-8 |
| Meta-analysis | D2 | Report the statistical methods used for meta-analyses, if performed. | 7-8 |
| Additional analyses | 16 | Describe methods of additional analyses (e.g., sensitivity or subgroup analyses, meta-regression), if done, indicating which were pre-specified. | 7-8 |
| RESULTS | | | |
| Study selection | 17 | Provide numbers of studies screened, assessed for eligibility, included in the review (and included in meta-analysis, if applicable) with reasons for exclusions at each stage, ideally with a flow diagram | 8 |
| Study characteristics | 18 | For each included study provide citations and present key characteristics including: a) participant characteristics (presentation, prior testing), b) clinical setting, c) study design, d) target condition definition, e) index test, f) reference standard, g) sample size, h) funding sources | 8-11 |
| Risk of bias and applicability | 19 | Present evaluation of risk of bias and concerns regarding applicability for each study. | 11-12 |
| Results of individual studies | 20 | For each analysis in each study (e.g. unique combination of index test, reference standard, and positivity threshold) report 2x2 data (TP, FP, FN, TN) with estimates of diagnostic accuracy and confidence intervals, ideally with a forest or receiver operator characteristic (ROC) plot. | NA |
| Synthesis of results | 21 | Describe test accuracy, including variability; if meta-analysis was done, include results and confidence intervals | 12-14 |
| Additional analysis | 23 | Give results of additional analyses, if done (e.g., sensitivity or subgroup analyses, meta-regression; analysis of index test: failure rates, proportion of inconclusive results, adverse events). | 12-14 |
| DISCUSSION | | | |
| Summary of evidence | 24 | Summarize the main findings including the strength of evidence | 15-17 |
| Limitations | 25 | Discuss limitations from included studies (e.g. risk of bias and concerns regarding applicability) and from the review process (e.g. incomplete retrieval of identified research). | 15-17 |
| Conclusions | 26 | Provide a general interpretation of the results in the context of other evidence. Discuss implications for future research and clinical practice (e.g. the intended use and clinical role of the index test) | 15-17 |
| OTHER | | | |
| Funding | 27 | For the systematic review, describe the sources of funding and other support and the role of the funders | 17 |

Adapted From: McInnes MDF, Moher D, et al. The PRISMA-DTA Group (2018). Preferred Reporting Items for a Systematic Review and Meta-analysis of Diagnostic Test Accuracy Studies: The PRISMA-DTA Statement. JAMA. 2018 Jan 23;319(4):388-396. doi: 10.1001/jama.2017.19163.

**Table S3. Keywords and search results in different database**

| **Database** | **Keyword** | **Date** | **Results** |
| --- | --- | --- | --- |
| PubMed | (Meningioma) AND (MRI imaging OR magnetic resonance imaging) AND (deep learning OR convolutional network OR machine learning OR artificial intelligence) AND (delineation OR segmentation) | 2023/12/20 | 66 |
| Embase | (Meningioma) AND (MRI imaging OR magnetic resonance imaging) AND (deep learning OR convolutional network OR machine learning OR artificial intelligence) AND (delineation OR segmentation) | 2023/12/20 | 115 |
| Web of Science | (Meningioma) AND (MRI imaging OR magnetic resonance imaging) AND (deep learning OR convolutional network OR machine learning OR artificial intelligence) AND (delineation OR segmentation) | 2023/12/20 | 140 |

**Table S4. Excluded article and reason.**

| Title | Exclusion reason |
| --- | --- |
| Brain tumor segmentation by cascaded multiscale multitask learning framework based on feature aggregation [24] | Outcome insufficient for quantitative meta-analysis |
| Meta-heuristic-based FCM-UNet segmentation with multi-objective function and deep learning for brain tumour classification [25] | Outcome not relate to interest |
| Efficient simultaneous segmentation and classification of brain tumors from MRI scans using deep learning [26] | Outcome insufficient for quantitative meta-analysis |
| Brain Tumor Segmentation Using Deep Learning on MRI Images [27] | Not meningioma |
| Automated segmentation of meningioma from contrast-enhanced T1-weighted MRI images in a case series using a marker-controlled watershed segmentation and fuzzy C-means clustering machine learning algorithm [28] | Not deep learning |
| Deep learning based semantic segmentation approach for automatic detection of brain tumor[29] | Outcome not relate to interest |
| Federated Learning: A Cross-Institutional Feasibility Study of Deep Learning Based Intracranial Tumor Delineation Framework for Stereotactic Radiosurgery [30] | Not meningioma |
| TAGU-Net: Transformer Convolution Hybrid-Based U-Net With Attention Gate for Atypical Meningioma Segmentation [31] | Outcome insufficient for quantitative meta-analysis |
| Deep Learning-Assisted Segmentation and Classification of Brain Tumor Types on Magnetic Resonance and Surgical Microscope Images [32] | Outcome insufficient for quantitative meta-analysis |
| Brain tumor segmentation and classification using hybrid deep CNN with LuNetClassifier [33] | Outcome not relate to interest |
| Edge U-Net: Brain tumor segmentation using MRI based on deep U-Net model with boundary information [34] | Outcome insufficient for quantitative meta-analysis |
| Detection of brain lesion location in MRI images using convolutional neural network and robust PCA [35] | Outcome insufficient for quantitative meta-analysis |
| A Brain Tumor Image Segmentation Method Based on Quantum Entanglement and Wormhole Behaved Particle Swarm Optimization [36] | Outcome not relate to interest |
| A Hybrid Approach Based on Deep CNN and Machine Learning Classifiers for the Tumor Segmentation and Classification in Brain MRI [37] | Retracted |
| Automated Detection and Classification of Meningioma Tumor from MR Images Using Sea Lion Optimization and Deep Learning Models [38] | Outcome insufficient for quantitative meta-analysis |
| Efficient deep learning models for brain tumor detection with segmentation and data augmentation techniques [39] | Outcome not relate to interest |
| Systematic Clinical Evaluation of a Deep Learning Method for Medical Image Segmentation: Radiosurgery Application [40] | Outcome not relate to interest |
| Multi Class Brain Tumor Segmentation Based On K-Means Clustering Technique [41] | Not deep learning |
| A dual-branch hybrid dilated CNN model for the AI-assisted segmentation of meningiomas in MR images [42] | Outcome insufficient for quantitative meta-analysis |
| FULLY AUTOMATED SEGMENTATION AND VOLUMETRIC MEASUREMENT OF INTRACRANIAL MENINGIOMA USING DEEP LEARNING [43] | Repeated article |
| A Hybrid Approach Based on Deep CNN and Machine Learning Classifiers for the Tumor Segmentation and Classification in Brain MRI [44] | Retracted |
| IIMFCBM: Intelligent Integrated Model for Feature Extraction and Classification of Brain Tumors Using MRI Clinical Imaging Data in IoT-Healthcare [45] | Outcome not relate to interest |
| Deep learning for automatic brain tumour segmentation on MRI: evaluation of recommended reporting criteria via a reproduction and replication study [46] | Outcome not relate to interest |
| Integrating anisotropic filtering, level set methods and convolutional neural networks for fully automatic segmentation of brain tumors in magnetic resonance imaging [47] | Outcome insufficient for quantitative meta-analysis |
| Enhanced deep-joint segmentation with deep learning networks of glioma tumor for multi-grade classification using MR images [48] | Not meningioma |
| FULLY AUTOMATIC MENINGIOMA SEGMENTATION USING T1-WEIGHTED CONTRAST-ENHANCED MR IMAGES ONLY [49] | Outcome insufficient for quantitative meta-analysis |
| Deep neural networks allow expert-level brain meningioma segmentation and present potential for improvement of clinical practice [50] | Outcome insufficient for quantitative meta-analysis |
| A Deep Learning Architecture for Meningioma Brain Tumor Detection and Segmentation [51] | Outcome not relate to interest |
| Deep Learning-Based Segmentation of Various Brain Lesions for Radiosurgery [52] | Outcome insufficient for quantitative meta-analysis |
| A semi-symmetric domain adaptation network based on multi-level adversarial features for meningioma segmentation [53] | Outcome insufficient for quantitative meta-analysis |
| A fuzzy logic-based meningioma tumor detection in magnetic resonance brain images usingCANFISandU-Net CNNclassification [54] | Outcome not relate to interest |
| Hybrid WCA–SCA and modified FRFCM technique for enhancement and segmentation of brain tumor from magnetic resonance images [55] | Outcome not relate to interest |
| Randomized multi-reader evaluation of automated detection and segmentation of brain tumors in stereotactic radiosurgery with deep neural networks [56] | Outcome not relate to interest |
| A Systematic Approach for MRI Brain Tumor Localization and Segmentation Using Deep Learning and Active Contouring [57] | Outcome not relate to interest |
| Application of deep learning for automatic segmentation of brain tumors on magnetic resonance imaging: a heuristic approach in the clinical scenario [58] | Outcome insufficient for quantitative meta-analysis |
| Automatic Meningioma Segmentation and Grading Prediction: A Hybrid Deep-Learning Method [59] | Repeated dataset |
| Fast meningioma segmentation in T1-weighted magnetic resonance imaging volumes using a lightweight 3D deep learning architecture [60] | Repeated dataset |
| Meningioma Segmentation in T1-Weighted MRI Leveraging Global Context and Attention Mechanisms [61] | Repeated dataset |
| Glioblastoma surgery imaging–reporting and data system: Validation and performance of the automated segmentation task [62] | Not meningioma |
| Deep neural networks allow expert-level brain meningioma detection, segmentation and improvement of current clinical practice [63] | Outcome insufficient for quantitative meta-analysis |
| A dual autoencoder and singular value decomposition based feature optimization for the segmentation of brain tumor from MRI images [64] | Outcome insufficient for quantitative meta-analysis |
| Automated meningioma detection and segmentation using deep neural networks [65] | Outcome insufficient for quantitative meta-analysis |
| Eye Tracking for Deep Learning Segmentation Using Convolutional Neural Networks [66] | Outcome not relate to interest |
| Fully automated detection and segmentation of meningiomas using deep learning on routine multiparametric MRI [67] | Repeated dataset |
| Evaluation of automated meningioma segmentation using deep-learning on multiparametric mri [68] | Repeated dataset |
| Multi-channeled MR brain image segmentation: A new automated approach combining BAT and clustering technique for better identification of heterogeneous tumors [69] | Not deep learning |
| Automatic segmentation of a meningioma using a computational technique in magnetic resonance imaging [70] | Not deep learning |
| Performance of a state-of-the art deep learning model for automated detection and segmentation of biopsy-proven meningiomas using multiparametric MRI [71] | Repeated dataset |
|  |  |

**Table S5. Quality assessment according to the Quality Assessment of Diagnostic Accuracy Studies 2 (QUADAS-2) criteria**

| **Source** | **Risk of bias** | | | | | | | | | | | **Concern of applicability** | | |
| --- | --- | --- | --- | --- | --- | --- | --- | --- | --- | --- | --- | --- | --- | --- |
|  | **Patient selection:** | | | **INDEX TEST** | | **Reference Standard** | | **Flow and Timing** | | | | **Patient selection** | **INDEX TEST** | **Reference Standard** |
|  | **Consecutive** | **Case- control** | **Inappropriate exclusions** | **Blind to reference standard** | **Threshold prespecified** | **Correctly classify the target condition** | **Blind to index test** | **Appropriate interval** | **Receive a reference standard** | **Same reference standard** | **All patients analyzed** |  |  |  |
| Lee et al. (2023) [72] | Unclear | No | No | Yes | Yes | Yes | Yes | Yes | Yes | Yes | Yes | Unclear | Low | Low |
| Kang et al. (2023) [73] | Yes | No | No | Yes | Yes | Yes | Yes | Yes | Yes | Yes | Yes | Low | Low | Low |
| Jun et al. (2023) [74] | Yes | No | No | Yes | Yes | Yes | Yes | Yes | Yes | Yes | Yes | Low | Low | Low |
| Dong et al. (2023) [75] | Yes | No | No | Yes | Yes | Yes | Yes | Yes | Yes | Yes | Yes | Low | Low | Low |
| Chen et al. (2023) [76] | Yes | No | No | Yes | Yes | Yes | Yes | Yes | Yes | Yes | Yes | Low | Low | Low |
| Huang et al. (2022) [77] | Yes | No | No | Yes | Yes | Yes | Yes | Yes | Yes | Yes | Unclear | Low | Low | Low |
| Chen et al. (2022) [78] | Yes | No | No | Yes | Yes | Yes | Yes | Yes | Yes | Yes | Yes | Low | Low | Low |
| Bouget et al. (2022) [79] | Yes | No | No | Yes | Yes | Yes | Yes | Yes | Yes | Yes | Unclear | Low | Low | Low |
| Lau kamp et al. (2022) [80] | Yes | No | No | Yes | Yes | Yes | Yes | Yes | Yes | Yes | Yes | Low | Low | Low |

**Table S6. The Checklist for Artificial Intelligence in Medical Imaging scores.**

| **Source** | **Title/Abstract** | **Introduction** | **Methods** | | | | | | | **Results** | | **Discussion** | **Other Information** | **Total Score** |
| --- | --- | --- | --- | --- | --- | --- | --- | --- | --- | --- | --- | --- | --- | --- |
|  |  |  | **Study design** | **Data** | **Ground truth** | **Data preparation** | **Model** | **Training** | **Evaluation** | **Data** | **Model performance** |  |  |  |
|  | **(2)** | **(2)** | **(2)** | **(7)** | **(5)** | **(3)** | **(3)** | **(3)** | **(5)** | **(2)** | **(3)** | **(2)** | **(3)** | **(42)** |
| Lee et al. (2023) [72] | 2 | 2 | 2 | 5 | 3 | 2 | 2 | 2 | 5 | 0 | 2 | 2 | 1 | 30 |
| Kang et al. (2023) [73] | 2 | 2 | 2 | 5 | 4 | 2 | 2 | 2 | 5 | 2 | 3 | 2 | 2 | 35 |
| Jun et al. (2023) [74] | 2 | 2 | 2 | 6 | 4 | 2 | 3 | 2 | 5 | 2 | 3 | 2 | 1 | 36 |
| Dong et al. (2023) [75] | 2 | 2 | 2 | 4 | 3 | 2 | 2 | 2 | 4 | 0 | 2 | 2 | 1 | 28 |
| Chen et al. (2023) [76] | 1 | 2 | 2 | 4 | 4 | 2 | 1 | 1 | 5 | 1 | 2 | 2 | 1 | 28 |
| Huang et al. (2022) [77] | 1 | 2 | 2 | 4 | 3 | 2 | 1 | 1 | 4 | 0 | 2 | 2 | 0 | 24 |
| Chen et al. (2022) [78] | 2 | 2 | 2 | 6 | 5 | 2 | 2 | 2 | 5 | 2 | 3 | 2 | 1 | 36 |
| Bouget et al. (2022) [79] | 1 | 2 | 2 | 3 | 0 | 2 | 2 | 2 | 3 | 0 | 2 | 2 | 1 | 22 |
| Lau kamp et al. (2022) [80] | 1 | 2 | 2 | 5 | 4 | 2 | 1 | 1 | 4 | 1 | 2 | 2 | 0 | 27 |
